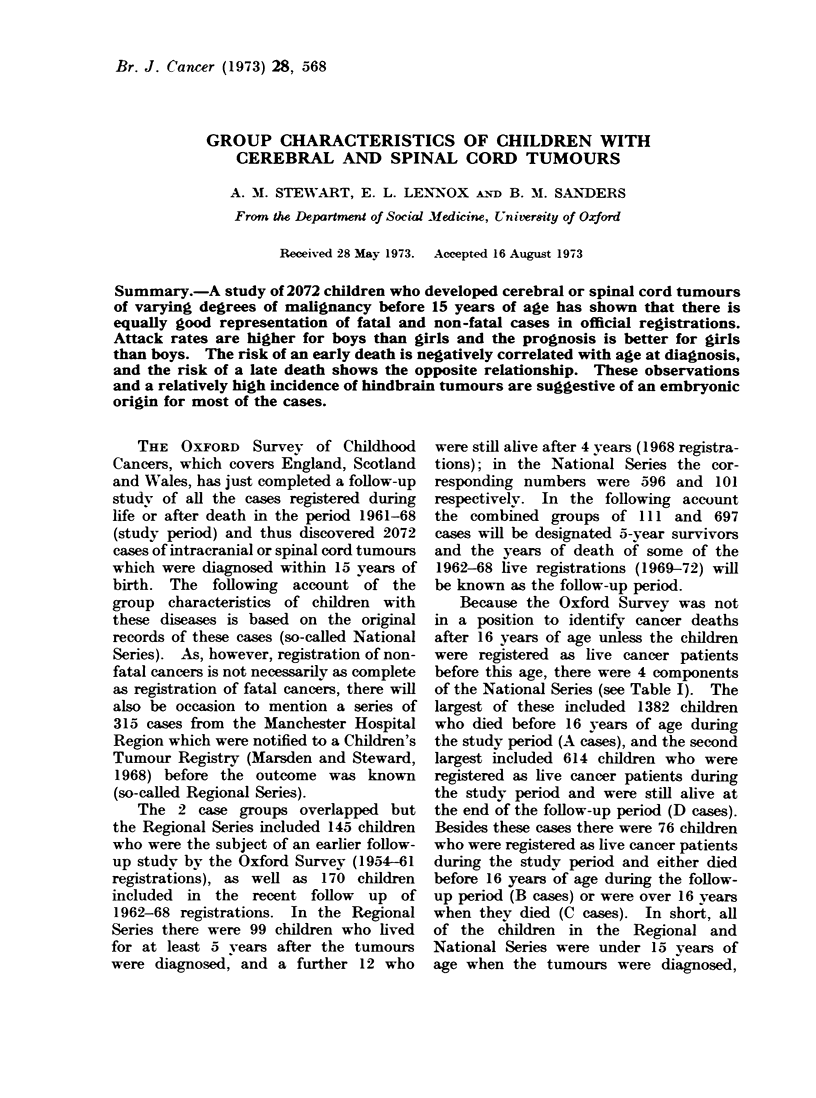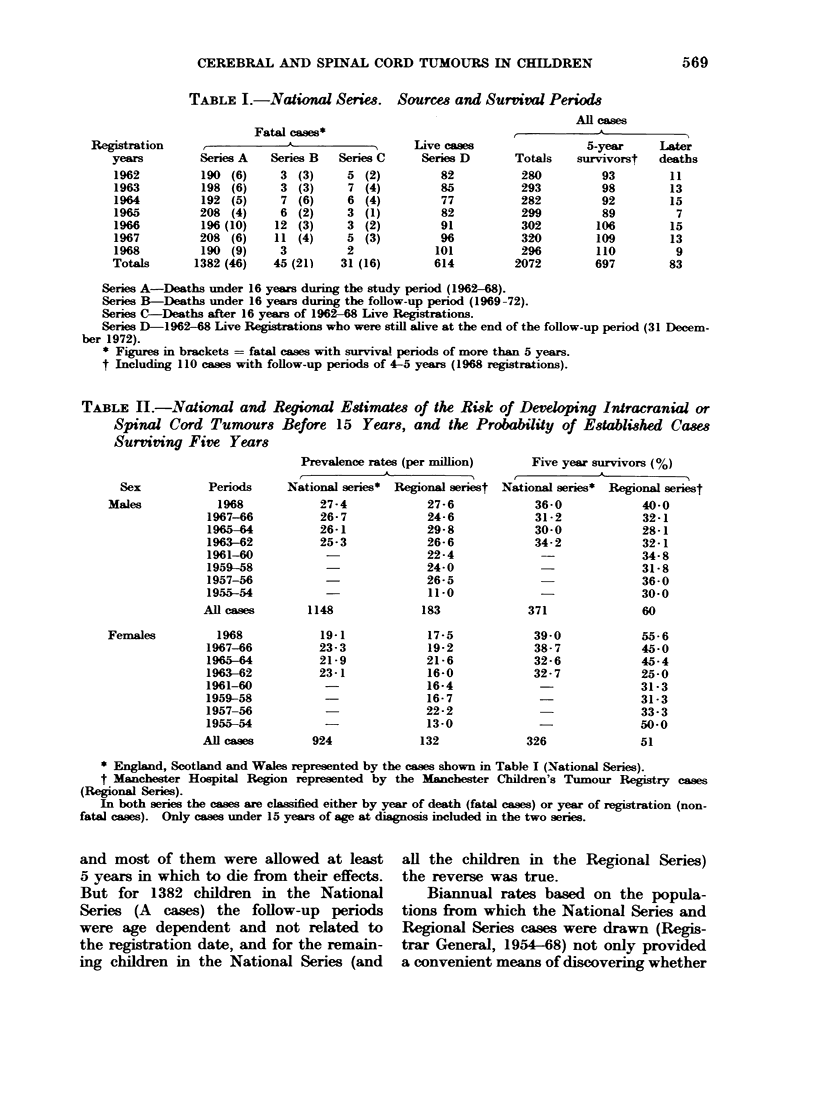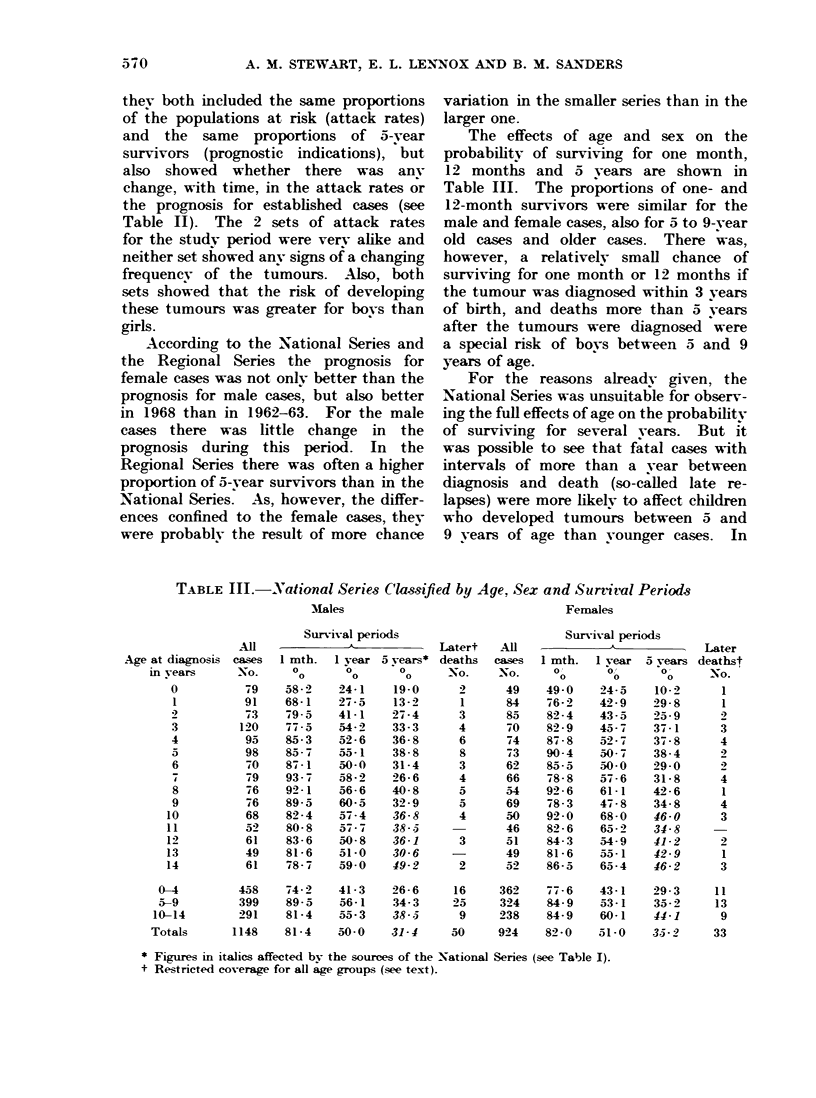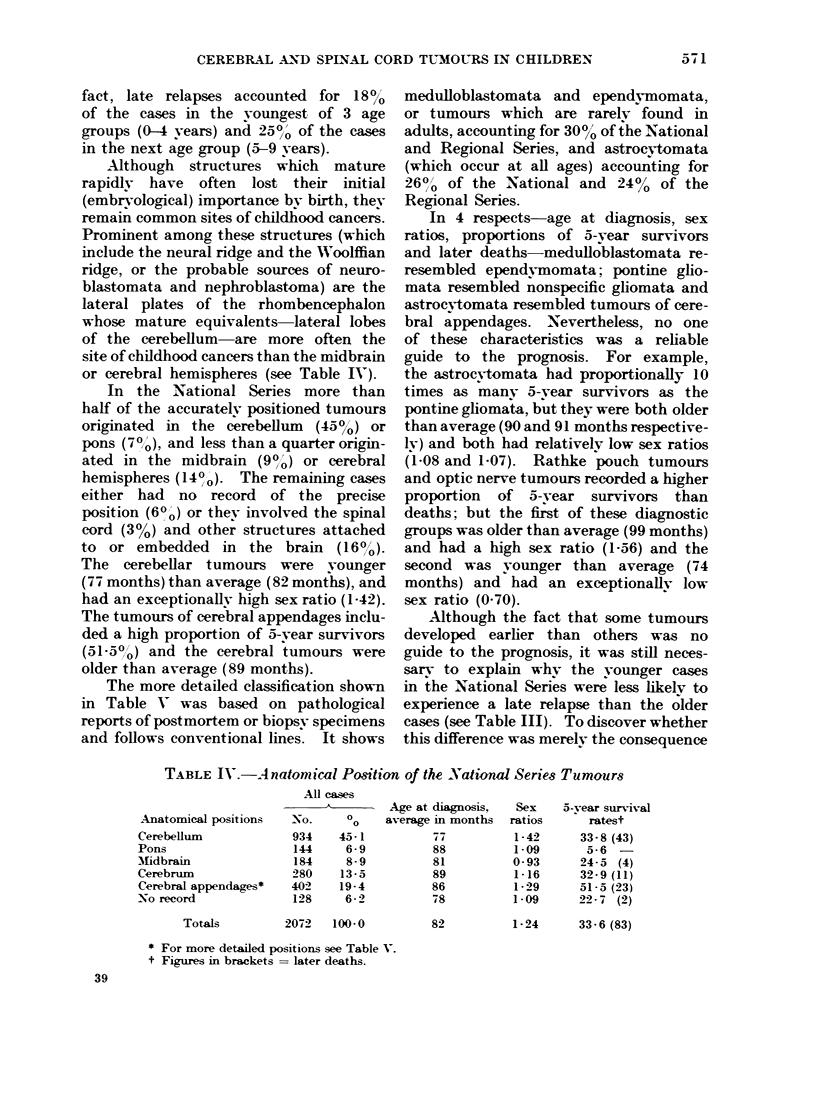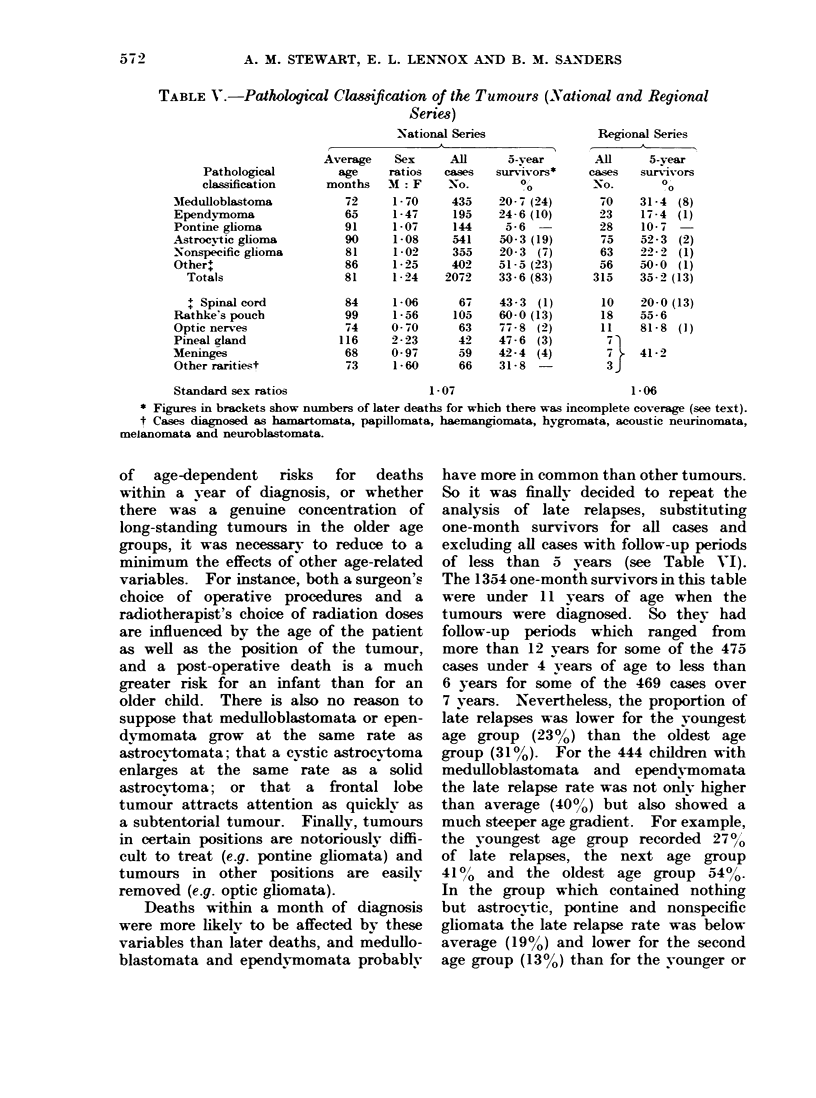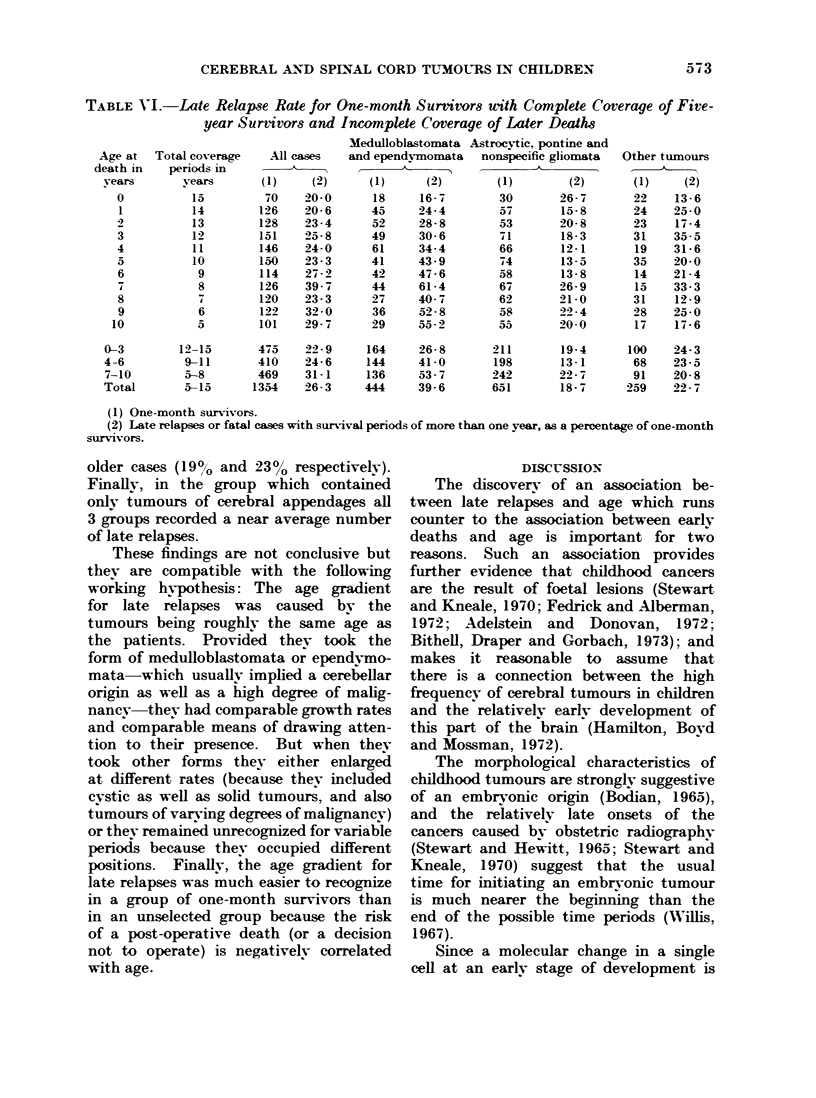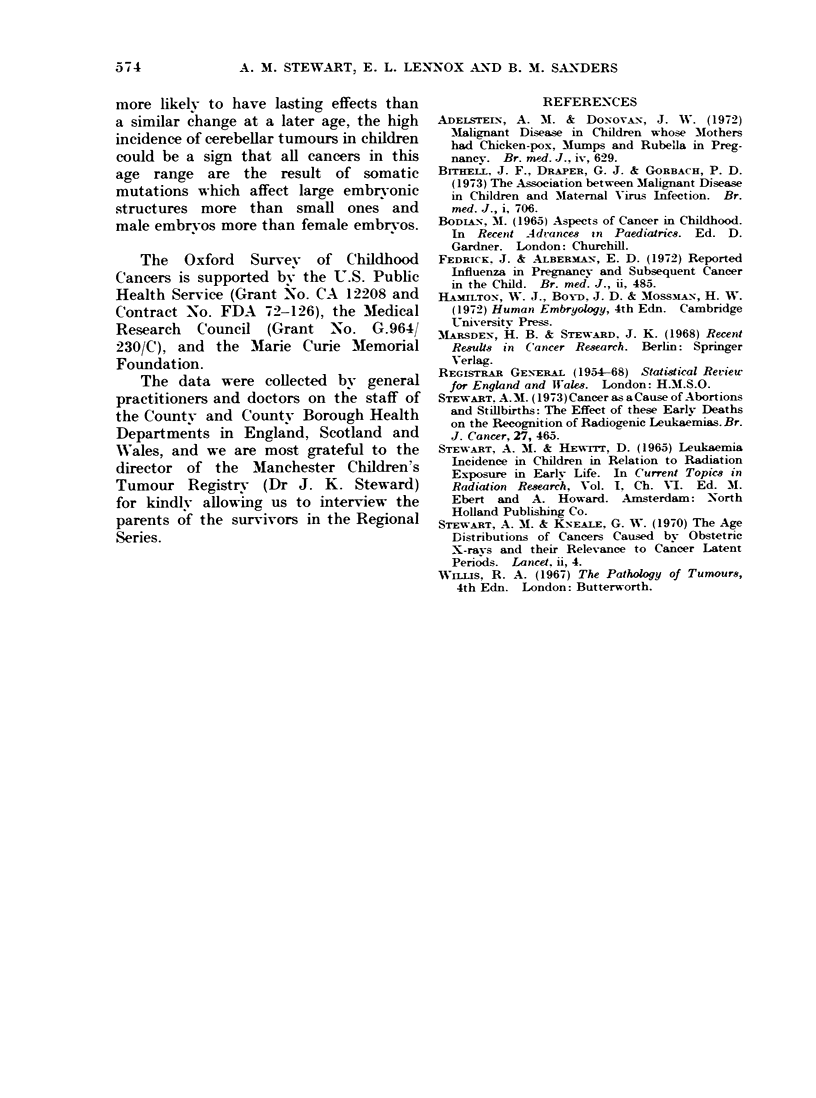# Group Characteristics of Children with Cerebral and Spinal Cord Tumours

**DOI:** 10.1038/bjc.1973.187

**Published:** 1973-12

**Authors:** A. M. Stewart, E. L. Lennox, B. M. Sanders

## Abstract

A study of 2072 children who developed cerebral or spinal cord tumours of varying degrees of malignancy before 15 years of age has shown that there is equally good representation of fatal and non-fatal cases in official registrations. Attack rates are higher for boys than girls and the prognosis is better for girls than boys. The risk of an early death is negatively correlated with age at diagnosis, and the risk of a late death shows the opposite relationship. These observations and a relatively high incidence of hindbrain tumours are suggestive of an embryonic origin for most of the cases.


Br. J. Cancer (1973) 28, 568

### GROUP CHARACTERISTICS OF CHILDREN WITH CEREBRAL AND SPINAL CORD TUMOURS

A. M. STEWART, E. L. LENNOX AND B. M. SANDERS

*From the Department of Social Medicine, University of Oxford*

Received 28 May 1973. Accepted 16 August 1973

**Summary.—A study of 2072 children who developed cerebral or spinal cord tumours of varying degrees of malignancy before 15 years of age has shown that there is equally good representation of fatal and non-fatal cases in official registrations. Attack rates are higher for boys than girls and the prognosis is better for girls than boys. The risk of an early death is negatively correlated with age at diagnosis, and the risk of a late death shows the opposite relationship. These observations and a relatively high incidence of hindbrain tumours are suggestive of an embryonic origin for most of the cases.**

THE OXFORD Survey of Childhood Cancers, which covers England, Scotland and Wales, has just completed a follow-up study of all the cases registered during life or after death in the period 1961–68 (study period) and thus discovered 2072 cases of intracranial or spinal cord tumours which were diagnosed within 15 years of birth. The following account of the group characteristics of children with these diseases is based on the original records of these cases (so-called National Series). As, however, registration of non-fatal cancers is not necessarily as complete as registration of fatal cancers, there will also be occasion to mention a series of 315 cases from the Manchester Hospital Region which were notified to a Children's Tumour Registry (Marsden and Steward, 1968) before the outcome was known (so-called Regional Series).

The 2 case groups overlapped but the Regional Series included 145 children who were the subject of an earlier follow-up study by the Oxford Survey (1954–61 registrations), as well as 170 children included in the recent follow up of 1962–68 registrations. In the Regional Series there were 99 children who lived for at least 5 years after the tumours were diagnosed, and a further 12 who were still alive after 4 years (1968 registrations); in the National Series the corresponding numbers were 596 and 101 respectively. In the following account the combined groups of 111 and 697 cases will be designated 5-year survivors and the years of death of some of the 1962–68 live registrations (1969–72) will be known as the follow-up period.

Because the Oxford Survey was not in a position to identify cancer deaths after 16 years of age unless the children were registered as live cancer patients before this age, there were 4 components of the National Series (see Table I). The largest of these included 1382 children who died before 16 years of age during the study period (A cases), and the second largest included 614 children who were registered as live cancer patients during the study period and were still alive at the end of the follow-up period (D cases). Besides these cases there were 76 children who were registered as live cancer patients during the study period and either died before 16 years of age during the follow-up period (B cases) or were over 16 years when they died (C cases). In short, all of the children in the Regional and National Series were under 15 years of age when the tumours were diagnosed,

TABLE I.—*National Series. Sources and Survival Periods*

| Registration years | Fatal cases* Series A | Series B | Series C | Live cases Series D | All cases Totals | 5-year survivors† | Later deaths |
|---|---|---|---|---|---|---|---|
| 1962 | 190 (6) | 3 (3) | 5 (2) | 82 | 280 | 93 | 11 |
| 1963 | 198 (6) | 3 (3) | 7 (4) | 85 | 293 | 98 | 13 |
| 1964 | 192 (5) | 7 (6) | 6 (4) | 77 | 282 | 92 | 15 |
| 1965 | 208 (4) | 6 (2) | 3 (1) | 82 | 299 | 89 | 7 |
| 1966 | 196 (10) | 12 (3) | 3 (2) | 91 | 302 | 106 | 15 |
| 1967 | 208 (6) | 11 (4) | 5 (3) | 96 | 320 | 109 | 13 |
| 1968 | 190 (9) | 3 | 2 | 101 | 296 | 110 | 9 |
| Totals | 1382 (46) | 45 (21) | 31 (16) | 614 | 2072 | 697 | 83 |

Series A—Deaths under 16 years during the study period (1962–68).
Series B—Deaths under 16 years during the follow-up period (1969-72).
Series C—Deaths after 16 years of 1962–68 Live Registrations.
Series D—1962–68 Live Registrations who were still alive at the end of the follow-up period (31 December 1972).
* Figures in brackets = fatal cases with survival periods of more than 5 years.
† Including 110 cases with follow-up periods of 4–5 years (1968 registrations).

TABLE II.—*National and Regional Estimates of the Risk of Developing Intracranial or Spinal Cord Tumours Before* 15 *Years, and the Probability of Established Cases Surviving Five Years*

| Sex | Periods | Prevalence rates (per million) National series* | Regional series† | Five year survivors (%) National series* | Regional series† |
|---|---|---|---|---|---|
| Males | 1968 | 27·4 | 27·6 | 36·0 | 40·0 |
| | 1967–66 | 26·7 | 24·6 | 31·2 | 32·1 |
| | 1965–64 | 26·1 | 29·8 | 30·0 | 28·1 |
| | 1963–62 | 25·3 | 26·6 | 34·2 | 32·1 |
| | 1961–60 | — | 22·4 | — | 34·8 |
| | 1959–58 | — | 24·0 | — | 31·8 |
| | 1957–56 | — | 26·5 | — | 36·0 |
| | 1955–54 | — | 11·0 | — | 30·0 |
| | All cases | 1148 | 183 | 371 | 60 |
| Females | 1968 | 19·1 | 17·5 | 39·0 | 55·6 |
| | 1967–66 | 23·3 | 19·2 | 38·7 | 45·0 |
| | 1965–64 | 21·9 | 21·6 | 32·6 | 45·4 |
| | 1963–62 | 23·1 | 16·0 | 32·7 | 25·0 |
| | 1961–60 | — | 16·4 | — | 31·3 |
| | 1959–58 | — | 16·7 | — | 31·3 |
| | 1957–56 | — | 22·2 | — | 33·3 |
| | 1955–54 | — | 13·0 | — | 50·0 |
| | All cases | 924 | 132 | 326 | 51 |

* England, Scotland and Wales represented by the cases shown in Table I (National Series).
† Manchester Hospital Region represented by the Manchester Children's Tumour Registry cases (Regional Series).
In both series the cases are classified either by year of death (fatal cases) or year of registration (non-fatal cases). Only cases under 15 years of age at diagnosis included in the two series.

and most of them were allowed at least 5 years in which to die from their effects. But for 1382 children in the National Series (A cases) the follow-up periods were age dependent and not related to the registration date, and for the remaining children in the National Series (and all the children in the Regional Series) the reverse was true.

Biannual rates based on the populations from which the National Series and Regional Series cases were drawn (Registrar General, 1954–68) not only provided a convenient means of discovering whether they both included the same proportions of the populations at risk (attack rates) and the same proportions of 5-year survivors (prognostic indications), but also showed whether there was any change, with time, in the attack rates or the prognosis for established cases (see Table II). The 2 sets of attack rates for the study period were very alike and neither set showed any signs of a changing frequency of the tumours. Also, both sets showed that the risk of developing these tumours was greater for boys than girls.

According to the National Series and the Regional Series the prognosis for female cases was not only better than the prognosis for male cases, but also better in 1968 than in 1962–63. For the male cases there was little change in the prognosis during this period. In the Regional Series there was often a higher proportion of 5-year survivors than in the National Series. As, however, the differences confined to the female cases, they were probably the result of more chance variation in the smaller series than in the larger one.

The effects of age and sex on the probability of surviving for one month, 12 months and 5 years are shown in Table III. The proportions of one- and 12-month survivors were similar for the male and female cases, also for 5 to 9-year old cases and older cases. There was, however, a relatively small chance of surviving for one month or 12 months if the tumour was diagnosed within 3 years of birth, and deaths more than 5 years after the tumours were diagnosed were a special risk of boys between 5 and 9 years of age.

For the reasons already given, the National Series was unsuitable for observing the full effects of age on the probability of surviving for several years. But it was possible to see that fatal cases with intervals of more than a year between diagnosis and death (so-called late relapses) were more likely to affect children who developed tumours between 5 and 9 years of age than younger cases. In

TABLE III.—*National Series Classified by Age, Sex and Survival Periods*

| | Males | | | | | Females | | | | |
|---|---|---|---|---|---|---|---|---|---|---|
| | | Survival periods | | | | | Survival periods | | | |
| Age at diagnosis in years | All cases No. | 1 mth. % | 1 year % | 5 years* % | Later† deaths No. | All cases No. | 1 mth. % | 1 year % | 5 years % | Later deaths† No. |
| 0 | 79 | 58·2 | 24·1 | 19·0 | 2 | 49 | 49·0 | 24·5 | 10·2 | 1 |
| 1 | 91 | 68·1 | 27·5 | 13·2 | 1 | 84 | 76·2 | 42·9 | 29·8 | 1 |
| 2 | 73 | 79·5 | 41·1 | 27·4 | 3 | 85 | 82·4 | 43·5 | 25·9 | 2 |
| 3 | 120 | 77·5 | 54·2 | 33·3 | 4 | 70 | 82·9 | 45·7 | 37·1 | 3 |
| 4 | 95 | 85·3 | 52·6 | 36·8 | 6 | 74 | 87·8 | 52·7 | 37·8 | 4 |
| 5 | 98 | 85·7 | 55·1 | 38·8 | 8 | 73 | 90·4 | 50·7 | 38·4 | 2 |
| 6 | 70 | 87·1 | 50·0 | 31·4 | 3 | 62 | 85·5 | 50·0 | 29·0 | 2 |
| 7 | 79 | 93·7 | 58·2 | 26·6 | 4 | 66 | 78·8 | 57·6 | 31·8 | 4 |
| 8 | 76 | 92·1 | 56·6 | 40·8 | 5 | 54 | 92·6 | 61·1 | 42·6 | 1 |
| 9 | 76 | 89·5 | 60·5 | 32·9 | 5 | 69 | 78·3 | 47·8 | 34·8 | 4 |
| 10 | 68 | 82·4 | 57·4 | *36·8* | 4 | 50 | 92·0 | 68·0 | *46·0* | 3 |
| 11 | 52 | 80·8 | 57·7 | *38·5* | — | 46 | 82·6 | 65·2 | *34·8* | — |
| 12 | 61 | 83·6 | 50·8 | *36·1* | 3 | 51 | 84·3 | 54·9 | *41·2* | 2 |
| 13 | 49 | 81·6 | 51·0 | *30·6* | — | 49 | 81·6 | 55·1 | *42·9* | 1 |
| 14 | 61 | 78·7 | 59·0 | *49·2* | 2 | 52 | 86·5 | 65·4 | *46·2* | 3 |
| 0–4 | 458 | 74·2 | 41·3 | 26·6 | 16 | 362 | 77·6 | 43·1 | 29·3 | 11 |
| 5–9 | 399 | 89·5 | 56·1 | 34·3 | 25 | 324 | 84·9 | 53·1 | 35·2 | 13 |
| 10–14 | 291 | 81·4 | 55·3 | *38·5* | 9 | 238 | 84·9 | 60·1 | *44·1* | 9 |
| Totals | 1148 | 81·4 | 50·0 | *31·4* | 50 | 924 | 82·0 | 51·0 | *35·2* | 33 |

* Figures in italics affected by the sources of the National Series (see Table I).
† Restricted coverage for all age groups (see text).

fact, late relapses accounted for 18% of the cases in the youngest of 3 age groups (0–4 years) and 25% of the cases in the next age group (5–9 years).

Although structures which mature rapidly have often lost their initial (embryological) importance by birth, they remain common sites of childhood cancers. Prominent among these structures (which include the neural ridge and the Woolffian ridge, or the probable sources of neuroblastomata and nephroblastoma) are the lateral plates of the rhombencephalon whose mature equivalents—lateral lobes of the cerebellum—are more often the site of childhood cancers than the midbrain or cerebral hemispheres (see Table IV).

In the National Series more than half of the accurately positioned tumours originated in the cerebellum (45%) or pons (7%), and less than a quarter originated in the midbrain (9%) or cerebral hemispheres (14%). The remaining cases either had no record of the precise position (6%) or they involved the spinal cord (3%) and other structures attached to or embedded in the brain (16%). The cerebellar tumours were younger (77 months) than average (82 months), and had an exceptionally high sex ratio (1·42). The tumours of cerebral appendages included a high proportion of 5-year survivors (51·5%) and the cerebral tumours were older than average (89 months).

The more detailed classification shown in Table V was based on pathological reports of postmortem or biopsy specimens and follows conventional lines. It shows medulloblastomata and ependymomata, or tumours which are rarely found in adults, accounting for 30% of the National and Regional Series, and astrocytomata (which occur at all ages) accounting for 26% of the National and 24% of the Regional Series.

In 4 respects—age at diagnosis, sex ratios, proportions of 5-year survivors and later deaths—medulloblastomata reresembled ependymomata; pontine gliomata resembled nonspecific gliomata and astrocytomata resembled tumours of cerebral appendages. Nevertheless, no one of these characteristics was a reliable guide to the prognosis. For example, the astrocytomata had proportionally 10 times as many 5-year survivors as the pontine gliomata, but they were both older than average (90 and 91 months respectively) and both had relatively low sex ratios (1·08 and 1·07). Rathke pouch tumours and optic nerve tumours recorded a higher proportion of 5-year survivors than deaths; but the first of these diagnostic groups was older than average (99 months) and had a high sex ratio (1·56) and the second was younger than average (74 months) and had an exceptionally low sex ratio (0·70).

Although the fact that some tumours developed earlier than others was no guide to the prognosis, it was still necessary to explain why the younger cases in the National Series were less likely to experience a late relapse than the older cases (see Table III). To discover whether this difference was merely the consequence

TABLE IV.—*Anatomical Position of the National Series Tumours*

| Anatomical positions | All cases No. | All cases % | Age at diagnosis, average in months | Sex ratios | 5-year survival rates† |
|---|---|---|---|---|---|
| Cerebellum | 934 | 45·1 | 77 | 1·42 | 33·8 (43) |
| Pons | 144 | 6·9 | 88 | 1·09 | 5·6 — |
| Midbrain | 184 | 8·9 | 81 | 0·93 | 24·5 (4) |
| Cerebrum | 280 | 13·5 | 89 | 1·16 | 32·9 (11) |
| Cerebral appendages* | 402 | 19·4 | 86 | 1·29 | 51·5 (23) |
| No record | 128 | 6·2 | 78 | 1·09 | 22·7 (2) |
| Totals | 2072 | 100·0 | 82 | 1·24 | 33·6 (83) |

* For more detailed positions see Table V.
† Figures in brackets = later deaths.

TABLE V.—*Pathological Classification of the Tumours (National and Regional Series)*

| | National Series | | | | Regional Series | |
|---|---|---|---|---|---|---|
| Pathological classification | Average age months | Sex ratios M : F | All cases No. | 5-year survivors* % | All cases No. | 5-year survivors % |
| Medulloblastoma | 72 | 1·70 | 435 | 20·7 (24) | 70 | 31·4 (8) |
| Ependymoma | 65 | 1·47 | 195 | 24·6 (10) | 23 | 17·4 (1) |
| Pontine glioma | 91 | 1·07 | 144 | 5·6 — | 28 | 10·7 — |
| Astrocytic glioma | 90 | 1·08 | 541 | 50·3 (19) | 75 | 52·3 (2) |
| Nonspecific glioma | 81 | 1·02 | 355 | 20·3 (7) | 63 | 22·2 (1) |
| Other‡ | 86 | 1·25 | 402 | 51·5 (23) | 56 | 50·0 (1) |
| Totals | 81 | 1·24 | 2072 | 33·6 (83) | 315 | 35·2 (13) |
| ‡ Spinal cord | 84 | 1·06 | 67 | 43·3 (1) | 10 | 20·0 (13) |
| Rathke's pouch | 99 | 1·56 | 105 | 60·0 (13) | 18 | 55·6 |
| Optic nerves | 74 | 0·70 | 63 | 77·8 (2) | 11 | 81·8 (1) |
| Pineal gland | 116 | 2·23 | 42 | 47·6 (3) | 7 | 41·2 (Pineal gland, Meninges, Other rarities combined) |
| Meninges | 68 | 0·97 | 59 | 42·4 (4) | 7 | |
| Other rarities† | 73 | 1·60 | 66 | 31·8 — | 3 | |
| Standard sex ratios | | | 1·07 | | | 1·06 |

* Figures in brackets show numbers of later deaths for which there was incomplete coverage (see text).
† Cases diagnosed as hamartomata, papillomata, haemangiomata, hygromata, acoustic neurinomata, melanomata and neuroblastomata.

of age-dependent risks for deaths within a year of diagnosis, or whether there was a genuine concentration of long-standing tumours in the older age groups, it was necessary to reduce to a minimum the effects of other age-related variables. For instance, both a surgeon's choice of operative procedures and a radiotherapist's choice of radiation doses are influenced by the age of the patient as well as the position of the tumour, and a post-operative death is a much greater risk for an infant than for an older child. There is also no reason to suppose that medulloblastomata or ependymomata grow at the same rate as astrocytomata; that a cystic astrocytoma enlarges at the same rate as a solid astrocytoma; or that a frontal lobe tumour attracts attention as quickly as a subtentorial tumour. Finally, tumours in certain positions are notoriously difficult to treat (*e.g.* pontine gliomata) and tumours in other positions are easily removed (*e.g.* optic gliomata).

Deaths within a month of diagnosis were more likely to be affected by these variables than later deaths, and medulloblastomata and ependymomata probably have more in common than other tumours. So it was finally decided to repeat the analysis of late relapses, substituting one-month survivors for all cases and excluding all cases with follow-up periods of less than 5 years (see Table VI). The 1354 one-month survivors in this table were under 11 years of age when the tumours were diagnosed. So they had follow-up periods which ranged from more than 12 years for some of the 475 cases under 4 years of age to less than 6 years for some of the 469 cases over 7 years. Nevertheless, the proportion of late relapses was lower for the youngest age group (23%) than the oldest age group (31%). For the 444 children with medulloblastomata and ependymomata the late relapse rate was not only higher than average (40%) but also showed a much steeper age gradient. For example, the youngest age group recorded 27% of late relapses, the next age group 41% and the oldest age group 54%. In the group which contained nothing but astrocytic, pontine and nonspecific gliomata the late relapse rate was below average (19%) and lower for the second age group (13%) than for the younger or

TABLE VI.—*Late Relapse Rate for One-month Survivors with Complete Coverage of Five-year Survivors and Incomplete Coverage of Later Deaths*

| Age at death in years | Total coverage periods in years | All cases (1) | All cases (2) | Medulloblastomata and ependymomata (1) | Medulloblastomata and ependymomata (2) | Astrocytic, pontine and nonspecific gliomata (1) | Astrocytic, pontine and nonspecific gliomata (2) | Other tumours (1) | Other tumours (2) |
|---|---|---|---|---|---|---|---|---|---|
| 0 | 15 | 70 | 20·0 | 18 | 16·7 | 30 | 26·7 | 22 | 13·6 |
| 1 | 14 | 126 | 20·6 | 45 | 24·4 | 57 | 15·8 | 24 | 25·0 |
| 2 | 13 | 128 | 23·4 | 52 | 28·8 | 53 | 20·8 | 23 | 17·4 |
| 3 | 12 | 151 | 25·8 | 49 | 30·6 | 71 | 18·3 | 31 | 35·5 |
| 4 | 11 | 146 | 24·0 | 61 | 34·4 | 66 | 12·1 | 19 | 31·6 |
| 5 | 10 | 150 | 23·3 | 41 | 43·9 | 74 | 13·5 | 35 | 20·0 |
| 6 | 9 | 114 | 27·2 | 42 | 47·6 | 58 | 13·8 | 14 | 21·4 |
| 7 | 8 | 126 | 39·7 | 44 | 61·4 | 67 | 26·9 | 15 | 33·3 |
| 8 | 7 | 120 | 23·3 | 27 | 40·7 | 62 | 21·0 | 31 | 12·9 |
| 9 | 6 | 122 | 32·0 | 36 | 52·8 | 58 | 22·4 | 28 | 25·0 |
| 10 | 5 | 101 | 29·7 | 29 | 55·2 | 55 | 20·0 | 17 | 17·6 |
| 0–3 | 12–15 | 475 | 22·9 | 164 | 26·8 | 211 | 19·4 | 100 | 24·3 |
| 4–6 | 9–11 | 410 | 24·6 | 144 | 41·0 | 198 | 13·1 | 68 | 23·5 |
| 7–10 | 5–8 | 469 | 31·1 | 136 | 53·7 | 242 | 22·7 | 91 | 20·8 |
| Total | 5–15 | 1354 | 26·3 | 444 | 39·6 | 651 | 18·7 | 259 | 22·7 |

(1) One-month survivors.
(2) Late relapses or fatal cases with survival periods of more than one year, as a percentage of one-month survivors.

older cases (19% and 23% respectively). Finally, in the group which contained only tumours of cerebral appendages all 3 groups recorded a near average number of late relapses.

These findings are not conclusive but they are compatible with the following working hypothesis: The age gradient for late relapses was caused by the tumours being roughly the same age as the patients. Provided they took the form of medulloblastomata or ependymomata—which usually implied a cerebellar origin as well as a high degree of malignancy—they had comparable growth rates and comparable means of drawing attention to their presence. But when they took other forms they either enlarged at different rates (because they included cystic as well as solid tumours, and also tumours of varying degrees of malignancy) or they remained unrecognized for variable periods because they occupied different positions. Finally, the age gradient for late relapses was much easier to recognize in a group of one-month survivors than in an unselected group because the risk of a post-operative death (or a decision not to operate) is negatively correlated with age.

### DISCUSSION

The discovery of an association between late relapses and age which runs counter to the association between early deaths and age is important for two reasons. Such an association provides further evidence that childhood cancers are the result of foetal lesions (Stewart and Kneale, 1970; Fedrick and Alberman, 1972; Adelstein and Donovan, 1972; Bithell, Draper and Gorbach, 1973); and makes it reasonable to assume that there is a connection between the high frequency of cerebral tumours in children and the relatively early development of this part of the brain (Hamilton, Boyd and Mossman, 1972).

The morphological characteristics of childhood tumours are strongly suggestive of an embryonic origin (Bodian, 1965), and the relatively late onsets of the cancers caused by obstetric radiography (Stewart and Hewitt, 1965; Stewart and Kneale, 1970) suggest that the usual time for initiating an embryonic tumour is much nearer the beginning than the end of the possible time periods (Willis, 1967).

Since a molecular change in a single cell at an early stage of development is more likely to have lasting effects than a similar change at a later age, the high incidence of cerebellar tumours in children could be a sign that all cancers in this age range are the result of somatic mutations which affect large embryonic structures more than small ones and male embryos more than female embryos.

The Oxford Survey of Childhood Cancers is supported by the U.S. Public Health Service (Grant No. CA 12208 and Contract No. FDA 72–126), the Medical Research Council (Grant No. G.964/230/C), and the Marie Curie Memorial Foundation.

The data were collected by general practitioners and doctors on the staff of the County and County Borough Health Departments in England, Scotland and Wales, and we are most grateful to the director of the Manchester Children's Tumour Registry (Dr J. K. Steward) for kindly allowing us to interview the parents of the survivors in the Regional Series.

### REFERENCES

ADELSTEIN, A. M. & DONOVAN, J. W. (1972) Malignant Disease in Children whose Mothers had Chicken-pox, Mumps and Rubella in Pregnancy. *Br. med. J.*, iv, 629.

BITHELL, J. F., DRAPER, G. J. & GORBACH, P. D. (1973) The Association between Malignant Disease in Children and Maternal Virus Infection. *Br. med. J.*, i, 706.

BODIAN, M. (1965) Aspects of Cancer in Childhood. In *Recent Advances in Paediatrics.* Ed. D. Gardner. London: Churchill.

FEDRICK, J. & ALBERMAN, E. D. (1972) Reported Influenza in Pregnancy and Subsequent Cancer in the Child. *Br. med. J.*, ii, 485.

HAMILTON, W. J., BOYD, J. D. & MOSSMAN, H. W. (1972) *Human Embryology*, 4th Edn. Cambridge University Press.

MARSDEN, H. B. & STEWARD, J. K. (1968) *Recent Results in Cancer Research.* Berlin: Springer Verlag.

REGISTRAR GENERAL (1954–68) *Statistical Review for England and Wales.* London: H.M.S.O.

STEWART, A.M. (1973) Cancer as a Cause of Abortions and Stillbirths: The Effect of these Early Deaths on the Recognition of Radiogenic Leukaemias. *Br. J. Cancer*, **27**, 465.

STEWART, A. M. & HEWITT, D. (1965) Leukaemia Incidence in Children in Relation to Radiation Exposure in Early Life. In *Current Topics in Radiation Research*, Vol. I, Ch. VI. Ed. M. Ebert and A. Howard. Amsterdam: North Holland Publishing Co.

STEWART, A. M. & KNEALE, G. W. (1970) The Age Distributions of Cancers Caused by Obstetric X-rays and their Relevance to Cancer Latent Periods. *Lancet*, ii, 4.

WILLIS, R. A. (1967) *The Pathology of Tumours*, 4th Edn. London: Butterworth.